# Chemical Profiling and Biological Activity of *Psydrax dicoccos* Gaertn

**DOI:** 10.3390/molecules28207101

**Published:** 2023-10-15

**Authors:** Kamaraj Veeramuthu, Vishal Ahuja, Pushparaj Annadurai, Daniel A. Gideon, Balamurugan Sundarrajan, Marius Emil Rusu, Vinothkanna Annadurai, Kandavel Dhandayuthapani

**Affiliations:** 1 Thanthai Periyar Government Arts and Science College (Autonomous), Bharathidasan University, Tiruchirappalli 620023, Tamil Nadu, India; kamaraj2275@gmail.com (K.V.); drsbalamurugan2008@gmail.com (B.S.); 2 University Institute of Biotechnology, Chandigarh University, Mohali 140413, Punjab, India; vahuja3@gmail.com; 3 University Centre for Research & Development, Chandigarh University, Mohali 140413, Punjab, India; 4 C.P.R. Environmental Education Center, 1 Eldams Road, Alwarpet, Chennai 600018, Tamil Nadu, India; pushpasaya10@gmail.com; 5 Department of Biochemistry, St. Joseph College, Bangalore 560025, Karnataka, India; dnlndrwster@gmail.com; 6 Department of Pharmaceutical Technology and Biopharmaceutics, Faculty of Pharmacy, Iuliu Hatieganu University of Medicine and Pharmacy, 400012 Cluj-Napoca, Romania; 7 School of Food and Biological Engineering, Jiangsu University, Zhenjiang 212013, China; a.vinothcellbio@gmail.com; 8 Department of Botany, Government Arts College for Men, Nandanam, University of Madras, Chennai 600035, Tamil Nadu, India

**Keywords:** *Psydrax dicoccos*, phytochemistry, antioxidant, cytotoxicity, anti-inflammatory, GC-MS, LC-MS, molecular docking, cancer

## Abstract

Breast cancer is one of the deadliest diseases in women with a mortality rate of 6.6%. Adverse effects of synthetic drugs have directed research toward safer alternatives such as natural compounds. This study focused on *Psydrax dicoccos* Gaertn, an evergreen tree abundantly distributed in Tamil Nadu (India) for its possible application against breast cancer cells. *P. dicoccos* leaf methanol extract, found within a wide range of phytochemicals, demonstrated cytotoxic effects against MCF7 breast cancer cells at IC_50_ of 34 μg/mL. The extract exhibited good antioxidant activities against DPPH^•^ (62%) and ABTS^•+^ (80%), as well as concentration-dependent (100–800 μg/mL) anti-inflammatory potential of 18–60% compared to standards, ascorbic acid or aspirin, respectively. Moreover, even low extract concentrations (10 μg/mL) inhibited the growth of *Escherichia coli* (1.9 ± 0.6 mm) and *Pseudomonas aeruginosa* (2.3 ± 0.7 mm), thus showing high antimicrobial and anti-inflammatory potential. GC-MS and LC-MS analyses identified 31 and 16 components, respectively, of which selected compounds were used to evaluate the interaction between key receptors (AKT-1, COX-2, and HER-2) of breast cancer based on binding energy (ΔG) and inhibition constant (K_i_). The results indicate that bioactive compounds from *P. dicoccos* have potential against breast cancer cells, but further evaluations are needed.

## 1. Introduction

Breast cancer is one of the deadliest, diseases in women with a high global incidence rate of 11.6% and a mortality rate of 6.6% [1]. Breast cancer can be categorized as (i) ERBB2/HER2 negative/hormone receptor-positive (~70% of all cases), (ii) ERBB2 positive (15–20% incidence), and (iii) triple-negative breast cancer (which lacks all three receptors, ERBB2, estrogen receptor and progesterone receptor). Protein kinase B (Akt1), which is a recurrently mutating somatic gene in breast, lung, and ovarian cancer, is a key factor in cancer cell survival, migration, and metastasis [2]. Oxidative stress-mediated inflammation and non-physiological levels of reactive oxygen species (ROS) can modulate the cellular physiology, gene expression and even metabolic pathways [3]. These factors might act as leading sources of cancer progression and survival. Inhibition of cell signaling pathways and quenching of oxidative stress can decrease cancer cell survival and promote apoptosis. Antioxidants and chemotherapeutic agents can both curb tumorigenesis and cancer proliferation via cell cycle arrest, induction of apoptosis, lowering inflammation and angiogenesis, and inhibition of metastasis [4]. Synthetic drugs and antibody–drug conjugates (e.g., becacizumab, transtuzumab) have been used for chemotherapy but can cause several adverse events such as pneumonitis, pulmonary fibrosis, febrile neutropenia, gastrointestinal complications, or neuropathies [5]. Owing to the toxicity of synthetic drugs, there has been a renewed interest in phytomedicine/traditional medicine to discover safer and novel plant metabolites that can inhibit breast cancer cell proliferation and progression [6].

Many in vitro and in vivo experiments have demonstrated the potential of plant compounds against several ailments [7], age-associated diseases [8], and even viral infections such as COVID-19 [9]. Secondary metabolites such as alkaloids, betalains, carotenoids, catechins, organosulfur compounds, phytosterols, and polyphenols/phenolic compounds have showed antioxidant and anti-inflammatory activities [10,11]. *Psydrax dicoccos* Gaertn., a plant in the family Rubiaceae found in tropical Asia, is widely distributed in the Pachamalai hills of Perambalur District, Tamil Nadu (India). Previously, the GC-MS (gas chromatography mass spectrophotometry) profile of the leaf extract was investigated, and its antifungal potential was evaluated [12]. In the present study, the work has been extended to assess its potential against breast cancer cells. Methanolic (MeOH) extract of *P. dicoccos* leaves was analyzed for anti-bacterial, antioxidant, cytotoxic, and anti-inflammatory effects. Phytochemicals were identified via GC-MS and LC-MS (liquid chromatography mass spectrophotometer), and further used for quantitative structure–activity relationship (QSAR)-based computer-aided screening and identification of specific targets against crucial receptors/enzymes involved in breast cancer survival and proliferation.

## 2. Results

### 2.1. Phytochemical Analysis

The MeOH extract of *P. dicoccos* leaves showed higher phytochemical diversity in comparison to acetone, ethanol, chloroform, and hexane (Table S1) and hence this extract was selected for bulk soxhletion and further experimental analyses. MeOH and aqueous extracts were found to possess the most diverse phytochemical compounds. The MeOH extract contained triterpenoids, sugars, flavonoids, tannins, amino acids, sterols, and carbohydrates. Since there was an intention to first identify the volatile compounds which may be present in the leaves, MeOH extraction was employed. Quantitative evaluation also suggested that MeOH extract possessed a substantial concentration of chlorophylls (14.48 mg/g) and carotenoids (2.18 mg/g), along with sugar (257 mg/g) and lipids (182 mg/g) (Table S2).

### 2.2. Antioxidant Activity of P. dicoccos Leaf Extract

**Figure 1:**
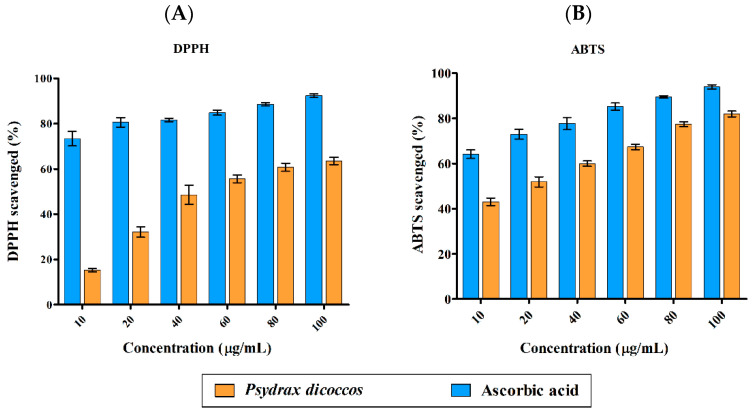
Antioxidant activity of *P. dicoccos* MeOH leaf extract: (**A**). DPPH radical-scavenging activity; (**B**). ABTS radical-scavenging activity. Both were assessed by comparing the efficacy of the plant extract with ascorbic acid as control at the same concentration range of 10–100 μg/mL.

DPPH radical (DPPH^•^)-scavenging activity was highly significant for all concentrations of ascorbic acid control (10–100 μg/mL) when compared to the plant extract (*p* < 0.001). A similar trend was also observed in ABTS radical (ABTS^•+^)-scavenging activity results. Antioxidant activity of the plant extract was in the range of <20–60% against DPPH radicals and 40–80% against ABTS radical cations (Figure 1A,B). The IC_50_ values for ABTS^•+^ and DPPH^•^ were 18 µg/mL and 44 µg/mL, respectively. Multifactor ANOVA determined that the p-values for DPPH^•^ and ABTS^•+^ were 0.012 and 0.004, which suggested that the model was significant, making ascorbic acid a more powerful antioxidant than the crude extract of the plant. This is often the case when a mixture of plant compounds are present, and only purified compounds may possess higher antioxidant activity relative to ascorbic acid control.

### 2.3. Anti-Bacterial Activity of P. dicoccos Leaf Extract

**Figure 2:**
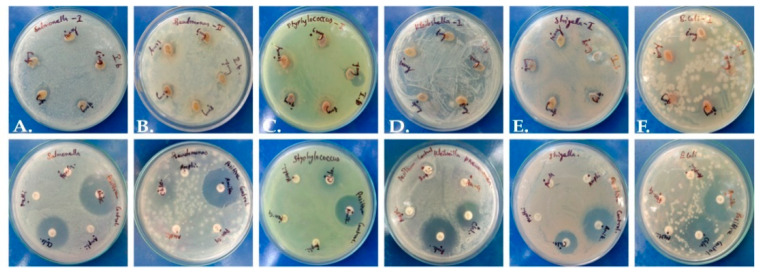
Antimicrobial activity of *P. dicoccos* leaf extract: (**A**)—*Salmonella*, (**B**)—*Pseudomonas*, (**C**)—*Staphylococcus*, (**D**)—*Klebsiella*, (**E**)—*Shigella* and (**F**)—*E. coli*.

The extract presented mild antimicrobial potential in comparison to standard antibiotic control discs (Figure 2). The zone of inhibition was merely 2 mm, while antibiotics yielded a zone around 11–12 mm (Table S3), and a maximum inhibitory zone of 2.3 mm was recorded against *Pseudomonas aeruginosa*. It is possible that a higher concentration of plant extract or the use of purified phytochemicals may have higher efficiency against pathogens.

### 2.4. Anti-Inflammatory Activity of P. dicoccos Leaf Extract

**Figure 3:**
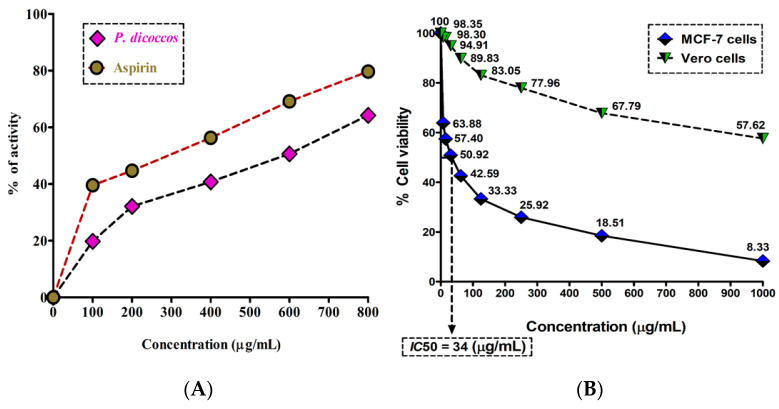
Anti-inflammatory and cytotoxic activity of *P. dicoccos* extract: (**A**). The HRBC membrane stabilization potential of *P. dicoccos* extract; (**B**) the effect of *P. dicoccos* extract on cell viability of MCF-7 cells (control: Vero cells).

Inflammation is one of the hallmarks of breast cancer pathogenesis and progression. It is mainly triggered by the conversion of arachidonic acid by cyclooxygenase-2 (COX-2) to form eicosanoids (e.g., prostaglandins and thromboxanes). These eicosanoids bring about the classic hallmarks of inflammation, such as erythema, edema, and pain [13]. The anti-inflammatory activity of *P. dicoccos* MeOH leaf extract was found to be appreciably higher when compared to the control anti-inflammatory drug aspirin (a COX-2 inhibitor) at comparable concentrations (100–800 μg/mL). Aspirin had 40–80% anti-inflammatory potential in comparison to the plant extract (20–60%) (Figure 3A). However, the activity of the plant extract may be amplified if purified compounds are used.

### 2.5. Cytotoxicity of P. dicoccos Leaf Extract

The MTT assay results demonstrated that the MeOH leaf extract of *P. dicoccos* was toxic to MCF-7 cells (IC_50_ = 34 μg/mL) but not to normal Vero cells (Figure 3B). In the context of breast cancer, it is well known that specific kinases such as HER2 receptor tyrosine kinase, PI3K, Akt, and mTOR are involved in breast cancer pathogenesis and progression [14]. 

Dysregulation/dysfunction of apoptosis and lapses in cell cycle checkpoint control are major causes of uncontrolled cancer cell proliferation and progression. Phytochemicals trigger apoptosis and curb the progression of tumor/cancer via antioxidant, anti-inflammatory, anti-proliferative, anti-angiogenic, anti-metastatic, proapoptotic, immunomodulatory, genoprotective, and pro-oxidant actions [15].

### 2.6. GC-MS and LC-MS Analyses of P. dicoccos Leaf Extract

**Figure 4:**
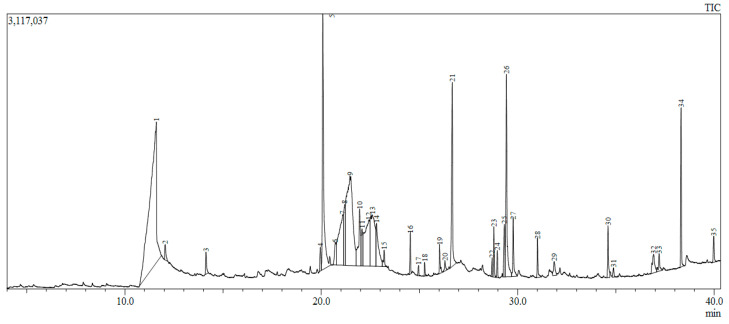
Gas chromatogram of methanolic plant extract.

Crude extracts possess an array of secondary metabolites and, hence, synergistic activities of many compounds may simultaneously target several key receptor targets. GC-MS chromatography identified 35 compounds mainly consisting of fatty acids, phenolics, sesquiterpenes (e.g., megastigmatrienone), phenolics (e.g., coumarins), glycosides, fatty acids (e.g., myristic acid), steroids (e.g., squalene), and δ-tocopherol classes (Figure 4; Table S4). Via LC-MS analysis, 15 compounds were identified; Table S5). Based on an extensive literature survey, three important target receptors—Akt1, COX2 and HER2—were chosen for molecular docking studies due to their importance in breast cancer progression. All the compounds from GC-MS and LC-MS were docked with receptors to find the binding affinity, and only the best energy compounds have been presented in this work. Only three compounds from GC-MS and two from LC-MS showed favorable interaction with the receptors and were considered for further analysis. 

### 2.7. Docking Results for Selected Compounds of P. dicoccos with Selected Breast Cancer Receptors

The selected compounds were evaluated for their interaction with target receptors in terms of K_i_ values and ligand efficiency (Table 1). 

Afatinib (AFT) and lapatinib (LTB) (with binding constants 45 and 138 Nm, respectively) exhibited higher affinity for Akt1 (PDB ID: 3CQW) in comparison to phytochemicals identified from the GC-MS spectrum. The other key compounds with favorable interactions were 2,3-Dihydro-benzofuran (DHB), megastigmatrienone (MST), and 2-Octylbenzoate (OBZ). From the compounds identified via LC-MS analysis, only Kaempferol (KMF) revealed favorable binding interaction with the receptors, while the others did not interact with any of the receptors. Inhibitory constant (K_i_) and binding energy (ΔG) were used as the main parameters for assessing the potential of the phytochemicals explored in the work. For Akt1, KMF was the most efficient among the four phytochemicals, but compared to the control drug molecules AFT and LTB, it was less efficient. For COX2, MST followed by KMF was not only efficient, but also superior to control drugs. For HER-2, OBZ, MST, and KMF performed well, but the control was found superior in interaction.

#### 2.7.1. Akt/Protein Kinase B

**Figure 5:**
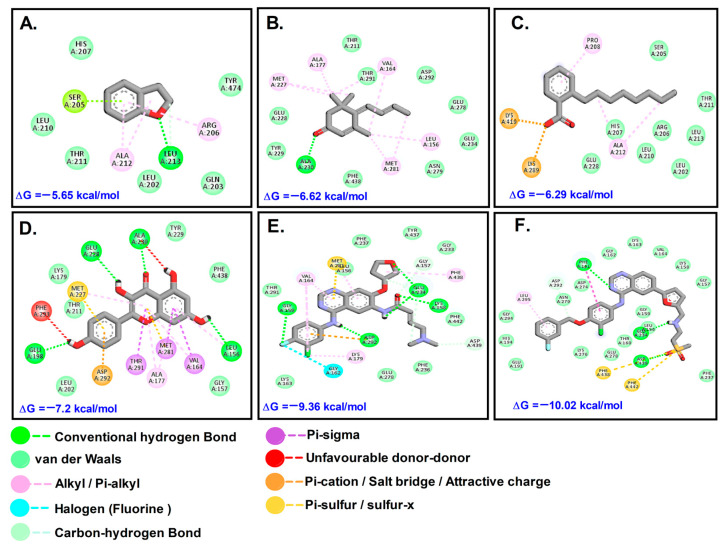
Molecular interactions with Akt1: (**A**). DHB; (**B**). MST; (**C**). OBZ; (**D**). KMF; (**E**). AFT; (**F**). LTB.

The docking results for Akt1 showed that the tested phytochemicals were not as effective as the control anti-cancer drugs LTB and AFT (Table 2, Figure 5). The test compounds docked to MMP9 at the canonical substrate binding site, which is like a ravine which contains an exposed catalytic Zn^2+^ atom. It was found that PTB bound to the active site via one of the channels; in contrast, OBZ bound to another side of MMP-9. MMP-9 is a key enzyme in the extracellular matrix of cancerous masses, as it cleaves several ECM substrates such as aggrecan, collagen, gelatin, and laminins, apart from metabolizing several non-ECM substrates such as angiotensin II, casein, plasminogen, and TGF-β1 [16]. This enables cancer cells to metastasize to other sites and establish secondary tumors and, hence, inhibition of MMP-9 can slow down metastasis. 

#### 2.7.2. COX-2

**Figure 6:**
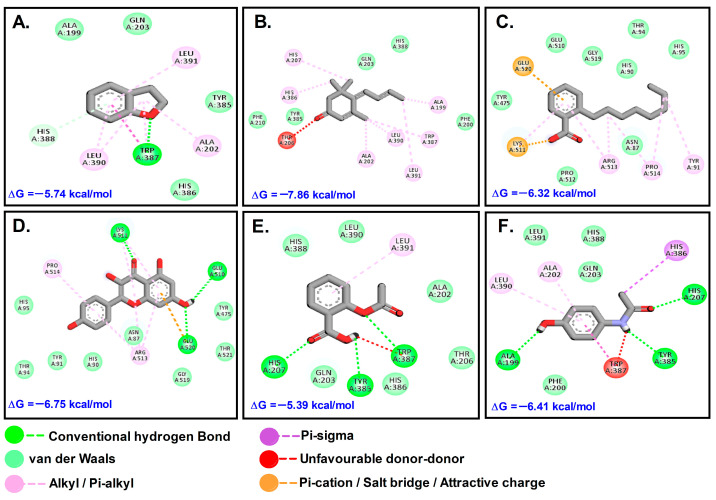
Molecular interactions with COX-2 (5F1A): (**A**). DHB; (**B**). MST; (**C**). OBZ; (**D**). KMF; (**E**). ASP; (**F**). PCM.

In comparison to ASP (−5 kcal/mol) and paracetamol (PCM) (−6 kcal/mol), MST had the most favorable binding to COX2 (ΔG = −7.86; K_i_ = 1.75 μM). KMF and MST were found to bind to the same binding site and have similar kind of interactions, while the other molecules such as DHB and OBZ docked at another site. The control compounds ASP and PCM were bound to the COX site (Table 3; Figure 6). 

#### 2.7.3. HER2

**Figure 7:**
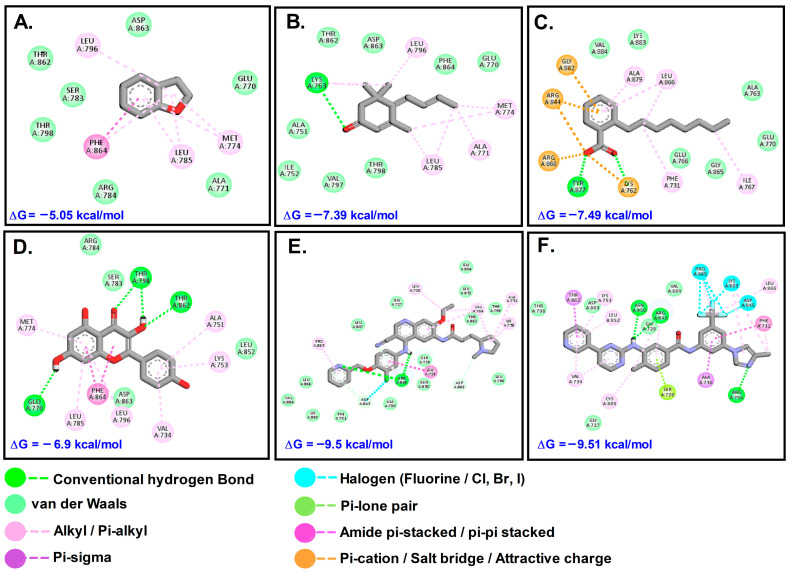
Interaction of target compounds with HER2: (**A**). DHB; (**B**). MST; (**C**). OBZ; (**D**). KMF; (**E**). PTB; (**F**). NTB.

The plant compounds were found to bind to two different pockets. Residues of HER2 which interacted with plant compounds as well as control drugs neratinib (NTB) and pyrotinib (PTB) are shown in Table 4. The interactions are presented in Figure 7. The binding energies of the control drugs PTB and NTB were the highest (−9.50 and −9.51) and were much better than those of the plant compounds. 

Among the receptors studied herein, phytochemicals had the most efficient interaction with the COX2 receptor. The binding energy of all four plant compounds were comparable to or lower than the standard drugs. For the AKT receptor, the binding efficiencies for the compounds were LTB > AFT > KMF > MST > OBZ > DHB. Similarly, the efficiencies for COX2 and HER2 receptors were MST > KMF > PCM > OBZ > ASP > DHB and NTB > PTB > OBZ > MST > KMF > DHB, respectively.

## 3. Discussion

In comparison to other solvents used for extraction, MeOH extract of *P. dicoccos* leaves was found to be enriched with phytochemicals, including triterpenoids, sugars, flavonoids, saponins, tannins, and sterols (Table S2). The total carotenoid content was found to be 2.18 mg/g, while sugar and total protein amounts were 257 mg/g and 1.2 mg/g, respectively. Lipid content was relatively high, with 182 mg/g, which translates to about 18.2%. Such a high lipid content may make this plant useful in biofuel production. The extract comprised 159 ± 2.8 mg/g flavonoids, 81.11 ± 1.1 mg/g phenolics, and 51.09 ± 2.2 mg/g tannins. The flavonoid content was substantially high, and these results show that this plant can be a source of potentially novel compounds. 

These phytochemicals are mainly responsible for the observed biological activities, including antimicrobial and antioxidant activity. Amalraj et al. [12] also reported that hydroalcoholic extract of *P. dicoccos* leaves comprised of 59.68 ± 0.3 mg GAE/g total phenolics, 57.85 ± 0.5 mg QRE/g total flavonoids, and 24.98 ± 0.17 mg AAE/g proanthocyanidin. GC-MS analysis identified 56 metabolites with squalene and cinnamic acid as important phytochemicals.

The methanolic crude extract contained triterpenoid, sugar, flavonoid, saponins, tannins, and sterols. Comparatively, the other solvents used for extraction, such as acetone, ethanol, chloroform and hexane, did not possess such a wide diversity of secondary metabolite classes (Table 1). Hence, methanol was used as the solvent for bulk soxhletion of the leaf powder to obtain a sizable quantity of the extract for further analyses. Crude plant extracts possess antimicrobial activity due to the presence of secondary metabolites, which aid in plant defense against pathogens. In one study, phytochemical screening of *P. dicoccos* confirmed the presence of carbohydrates, phenolics, flavonoids, glycosides, and tannins [17]. In another work, MeOH extract of *P. dicoccos* was found to possess antifungal activity against *Candida albicans*, *C. guilliermondii*, *C. glabrata*, *C. krusei*, *C. parapsilosis*, *C. tropicalis*, *Epidermophyton flocossum*, *Microsporum gypseum*, *Trichophyton rubrum*, and *T. mentagrophytes*. The zone of inhibition against fungal pathogens was 7–16 mm in diameter. The MIC and MFC values were 125–500 µg/mL and 250–1000 µg/mL, respectively. The GC-MS profile of methanol extract also confirmed the presence of benzofuran and n-hexadecanoic acid [18]. In a study, purified OBZ, a benzoic acid ester (from the essential oil of *Salvia urmiensis* Bunge) was shown to exhibit antimicrobial activity against a wide range of pathogenic strains [19]. 

Based on the GC-MS results, molecules of potential interest were selected after screening using AUTODOCK. Those compounds were docked to critically important breast cancer signaling targets such as HER2, COX2, and MMP-9, and the compounds with the lowest binding scores were chosen for comparison. In silico analysis aided in prediction of drug–target interactions and ADME assessments and, thereby, hastened the drug discovery process. Molecular docking is highly preferred in drug discovery to study the potential therapeutic targets and predict ligand–protein interactions. Qawoogha and Shahiwala [20] identified yuanhuanin, theaflavin, and genistein as potential phytochemicals with activity against colorectal cancer. Taher et al. [21] reported two new flavonols, namely quercetin-3-*o*-(glucopyranosyl 1→2 ribopyranoside) and kaempferol-3-*o*-(glucopyranosyl 1→2 ribopyranoside), from *Hymenosporum flavum* (Hook.) extract. In silico analysis revealed that the plant extract possessed cytotoxic effect against HepG2 cells, mainly by acting against RAF-1 and ERK-2. It was also suggested that the extract improved the effect of sorafenib in targeting both RAF-1 and ERK-2 pathways.

Because of their ability to quench free radicals such as ROS, reactive nitrogen species [22,23], reactive sulfur species (RSS), and reactive carbon-centered radicals, plant metabolites are known to prevent oxidative stress-induced cancer and slow down aging. The antioxidant activity of the extract was lower than that of the standard, ascorbic acid, in the range of 10–100 μg/mL. Ascorbic acid, being a pure compound (and as expected), had significantly higher antioxidant activity than the plant MeOH crude extract, which possessed a mixture of phytochemicals. The MeOH leaf extract exhibited good antioxidant activities against DPPH^•^ (62%) and ABTS^•+^ (80%). DPPH^•^, which is violet in color, reacts with antioxidant molecules, which donate a proton and an electron to a nitrogen atom. The color of the solution changes to yellow or colorless depending on the intensity of the antioxidant power. ABTS^•+^ is generated from ABTS overnight, and the radical solution is mixed with antioxidant and a similar reaction involving a nitrogen atom in the molecule (donation of proton and electron by antioxidant) leads to generation of ABTS and thus the disappearance of its bottle green color while simultaneously generating antioxidant radicals.

The anti-inflammatory activity of the *P. dicoccos* MeOH leaf extract was found to be appreciably higher when compared to the control anti-inflammatory drug aspirin (a COX2 inhibitor) at comparable concentrations (in the range of 100–800 μg/mL). While aspirin, being a pure compound, inhibited inflammation (in the order of 40–80%) by stabilizing human RBC membranes and thereby prevented hemolysis, the plant extract inhibited inflammation by 20–60% at the same concentration range (Figure 3A). A clearer picture can be obtained if purified compounds from the plant extract were to be studied in the future. 

Breast cancer is a complex and heterogeneous disease with significant differences in tissue markers and mechanisms of proliferation. Inflammation, oxidative stress, dysregulated cellular signal transduction pathways owing to gene mutations, epigenetic causes, and a plethora of other factors can determine the treatment modalities, as well as clinical endpoints/outcomes. A single plant metabolite may possess a combination of several beneficial properties such as antioxidant, antimicrobial, and anticancer, as well as anti-inflammatory activity. These overlapping activities can be envisaged to occur in a cellular milieu and, hence, these activities are intertwined and need not be presumed to be distinct properties of a given plant metabolite. 

In the context of breast cancer, it is well known that specific kinases such as HER2 receptor tyrosine kinase, PI3K, Akt and mTOR are involved in breast cancer pathogenesis and progression [14]. The signaling of mTOR is connected to activation of the Akt pathway. Numerous signaling pathways are triggered by multiple cell surfaces as well as nuclear receptor-based hormones, and environmental/extracellular factors can influence cancer cell signaling and survival. In all cancers, apoptosis is dysfunctional/lacking, and there are lapses in cell cycle checkpoint controls. Since phytochemicals have been demonstrated to trigger apoptosis, they have the ability to shrink tumors and curb the progression of cancer via their antioxidant, anti-inflammatory, anti-proliferative, anti-angiogenic, anti-metastatic, proapoptotic, immunomodulatory, genoprotective, and pro-oxidant actions [15]. Since crude extracts possess an array of different secondary metabolites, synergistic activities of many compounds may simultaneously target several key receptor targets. However, isolated/purified compounds can possess affinity for specific receptors/protein targets and thus inhibit cancer growth and trigger apoptosis. The IC_50_ value of phytochemicals present in *P. dicoccos* which could offer cytotoxic activity may need to be investigated in the future by using purified compounds and appropriate cellular models to establish their mechanisms of action. 

Compounds possessing the 2,3-dihydro-5-benzofuranol (DHB) ring have antioxidant properties and inhibit leukotriene biosynthesis in leukocytes. Also, the DHB ring is a template for the design of efficient lipooxygenase inhibitors [24]. That same work showed that 2,3-dihydro-5-benzofuranols exhibit dual inhibitory action against both LOX and COX enzymes and simultaneously showed substantial antioxidant activity. The docking study performed herein demonstrates that MST, DHB, and OBZ inhibit COX-2. The flavonoid aglycone KMF is a well-known natural compound with anti-inflammatory, antioxidant and anti-proliferative activity [25]. KMF inhibits NOS and COX-2; it also lowers C-reactive protein (CRP) levels and inhibits pro-inflammatory NFkB, which, in the presence of oxidative stress, upregulates the expression of antioxidant enzymes, which can further lead to cancer progression and chemoresistance [26]. 

Akt/protein kinase B is an important hub of cellular signaling [27,28] and is responsible for relaying the signals of growth factors/hormones. mTOR is downstream from Akt in the PI3K-Akt-mTOR pathway. Akt relays the phosphorylation signal-mediated activation of mTORC1, which enhances cancer cell survival [29].

Cyclooxygenase/prostaglandin H-synthase is an important enzyme responsible for metabolism of arachidonic acid and the synthesis of eicosanoids—prostaglandins and thromboxanes. COX-1 is constitutively expressed in most tissues/organs, while COX-2 is an inducible enzyme which is expressed in response to certain stimuli in mainly inflammatory cells and tissues [30]. In breast cancer, COX-2 plays a significant role in inflammation and is a crucial target of non-steroidal anti-inflammatory drugs (NSAIDs), such as aspirin (ASP) and ibuprofen, that may be chemoprotective agents against breast cancer [30]. Since COX-2 inhibitors are reported to be both chemoprotective and helpful in treatment of breast cancer [31], the COX-2 inhibitory potential (K_i_) of the phytochemicals selected for the study were compared to standard NSAID controls such as ibuprofen, aspirin and paracetamol. MST, KMF, OBZ, and DHB were found to bind to COX-2 at the COX site with appreciable binding energies and therefore the molecular docking study of COX-2 corroborated the in vitro anti-inflammatory activity study. In another study, Prathyusha et al. [32] evaluated the anti-ulcer potential of MeOH extract of *P. dicoccus* whole plant in a diabetic rat model. They prepared a methanolic extract of the plant via hot continuous extraction. The experimental animal group with *P. dicoccos* whole plant MeOH extract treatment protected against aspirin-induced ulcer in a concentration-dependent manner. The extract remarkably enhanced ulcer-protection (against untreated control) by 7.61% to 24.16%, at a concentration range of the extract between 200 mg/kg to 400 mg/kg. Besides anti-ulcer activity, the plant extract also showed appreciable antioxidant activity. The methanol extract of *P. dicoccus* whole plant did not exhibit any toxicity up to 2000 mg/kg body weight in Wistar rats. 

HER2 is the receptor for binding EDGF and is known to signal the effects of EDGF via its tyrosine kinase activity. When not directly bound to DNA, HER2 is known to activate PI3K and MAPK signaling cascades. HER2 activation results in concomitant triggering of the Ras-Raf-MEK-ERK pathway and PI3K-Akt-mTOR pathway, which is crucial for breast cancer cell proliferation, cancer survival, and cancer chemoresistance. HER2 signaling can be blocked by antagonists, provided that the inhibitors can bind to the ligand binding site. However, mutant HER2 can have alterations in their ligand binding pockets, or possess activity even in the absence of an inhibitory ligand due to gain of function mutations. At present, it is unclear whether the study compounds can serve as an inhibitor of HER2, as gain of function HER2 mutants can pose a significant problem in experimental therapeutics. In that case, if the test compounds can inhibit Akt, mTOR or any of the relay cascades which transduce ErbB/HER2 external growth factor stimuli. This is why Akt was explored in the docking studies. More experimental studies need to be performed in the future to assess whether these test compounds could mechanistically block mutant HER2 signaling. The docking scores of the test compounds, although not as attractive as the control compounds, were in the range of −6 to −7 for 3 of the 4 compounds which were docked to Akt. 

Taken together, the results from this work and the results from published literature reports suggest that certain phytochemical compounds present in *P. dicoccos* may be responsible for varied beneficial biological properties such as antioxidant, cytotoxic, antimicrobial, and anti-inflammatory effects. Together, these phytochemicals could synergistically act in milieu through a combination of the diverse activities discussed herein, to inhibit microbial growth and breast cancer cell proliferation. However, despite favorable in vitro and in silico outcomes, the safety level and the beneficial effects could only be determined via further in vivo toxicological studies [33]. 

## 4. Materials and Methods

### 4.1. Chemicals and Reagents

All the chemicals and reagents used were purchased from reputed vendors (SRL, India and HiMedia, Mumbai, India) and were of ultra-pure grade. Antibiotic discs were obtained from HiMedia and their Cat.Nos. are as follows: ampicillin 10 μg (SD002), amikacin 10 μg (SD082), chloramphenicol 10 μg (SD081), bacitracin 10 μg (SD105), methicillin 5 μg (SD137), and penicillin-G 30 μg (SD144). 

### 4.2. Plant Collection

*P. dicoccos* leaves were collected from the Pachamalai Hills, Tiruchirappalli District, Tamil Nadu, India (11.3258° N and 78.6308° E). The collected botanical sample (Specimen #VK001) was authenticated by Rapinat Herbarium, St. Joseph’s College, Tiruchirappalli (Figure S1). 

### 4.3. Phytoextraction

A wide range of solvents including aqueous, methanol, ethanol, acetone, chloroform, and hexane were used for extraction as per an established protocol [34]. Since the methanol extract had wider range of phytochemicals, it was used for further evaluation. Extract was dried and dissolved in either methanol or DMSO for further analysis.

### 4.4. Phytochemical Analysis

Various methods were employed for the quantitative estimation of *P. dicoccos* leaf extract. Arnon’s method was used to check the presence of chlorophyll and carotenoids [35,36]. The Dubois method was carried out to quantify the sugar content [37,38]. The gravimetric method was used to check the total lipid content [39,40]. The Lowry method was carried out to estimate protein [41,42]. The Troll and Cannan method was performed to check the total amino acid content [43]. The Folin–Ciocalteu method was followed to quantify the total phenols, flavonoids, and tannins [44,45].

### 4.5. Anti-Bacterial Activity

Anti-bacterial activity was determined via agar well diffusion [46,47]. Plant extract stock (10 mg/mL) was used in the concentration range of 6–10 mg/mL against microbial pathogens. Post-addition of extract, plates were incubated at 37 °C for 12 h. Commercially available antibiotic discs, amikacin (10 μg), chloramphenicol (10 μg), bacitracin (10 μg), penicillin (30 μg), and methicillin (5 μg), were taken as control. The protocol followed for antibacterial activity can be summarized as follows: overnight cultures of the strains were diluted in nutrient broth to 0.5 MacFarland units (1.5 × 10^8^ CFU/mL) and spread onto nutrient agar plates. Wells were formed using a sterile gel puncher, and antibiotic discs were placed as controls. The cultures were incubated at 37 °C overnight in a bacteriological incubator, and the zones of inhibition were measured on the following day using a ruler. For preparation of the inoculum, care was taken to conform to the guidelines of Wiegand et al. [48]. Other details related to methodology were followed as per our earlier published work [49].

### 4.6. Antioxidant Activity

#### 4.6.1. DPPH^• o^ Free Radical-Scavenging Assay

DPPH radical-scavenging activity was determined with the pre-established protocol [50]. The experiment was carried out by taking 1:1 of 10–100 μg/mL of leaf extract and DPPH methanol solution and comparing it with L-ascorbic acid as standard according to the protocol of Brand-Williams et al., 1995 [51]. The inhibition percentage was calculated using Equation (1):

Inhibition (%) = (Ac − As/Ac) × 100(1)

where Ac denotes absorbance of the control (L-ascorbic acid) and As denotes the absorbance of the sample.

#### 4.6.2. ABTS^•+^ Assay

Freshly prepared ABTS solution (7 mM) was mixed with 2.45 mM potassium persulfate (1:1 ratio) and incubated for 12–16 h at room temperature in the dark. Then, the plant extract was added to this solution (10–100 (g/mL), and the potential was compared with L-ascorbic acid [52]. The assay was monitored at 734 nm, and the assay was performed as per Re et al. [53]. The radical-scavenging activity (%) was calculated using Equation (1).

### 4.7. Anti-Inflammatory Activity of P. dicoccos Leaf Extract

Human red blood cell (HRBC) membrane stabilization study [54,55] was conducted to determine the anti-inflammatory potential of the extract. Healthy human blood samples were mixed with fresh Alsever solution as an anticoagulant (1:1). This mixture was centrifuged at 10,000× *g* rpm for 15 min, and the supernatant was discarded. Collected RBCs were washed and suspended in phosphate-buffered saline (PBS) (pH 7.3). Different concentrations of extract (100–800 μg/mL) were added to suspension, PBS, and hypo saline and kept at 37 °C for 30 min. Hemolysis was determined spectrophotometrically at 620 nm using Equation (2):

Hemolysis (%) = T/C × 100(2)

where, T is the Test sample, and C is the Control sample.

### 4.8. Cytotoxicity of P. dicoccos Leaf Extract

Cytotoxic activity of *P. dicoccos* extract was assessed against MCF-7 cell line via MTT assay [56,57]. MCF-7 cells were purchased from the National Centre for Cell Science (NCCS) in Pune, India, grown to confluence in DMEM medium with 10% fetal bovine serum (in the presence of 100 μg/mL of streptomycin and 50μg/mL of penicillin) and incubated in CO_2_ (5%) at room temperature (37 °C). MCF-7 cells (1 × 10^5^/well) were transferred to 24-well plates and mixed with various concentrations of *P. dicoccos* leaf extract and incubated with 5% CO_2_, at 37 °C for 24 hrs. Residual traces of plant extract were removed from the plates via gentle rinsing with PBS (pH 7.4). Cell viability was determined by 0.5% MTT using DMSO as blank. Cell viability was calculated by Equation (3):

Cell viability (%) = Treated cells/Control cells × 100(3)

IC_50_ (concentration of plant extract resulting in 50% inhibition of cell viability) was determined from the graphs using non-linear regression (log inhibitor vs. normalized response) option in GraphPad Prism 5.02.

### 4.9. GC-MS and LC-MS Profiling of the Methanol Leaf Extracts of P. dicoccos

The phytochemicals constituents in methanolic extract were identified using GC-MS (Perkin Elmer Clarus 500, Shelton, CT, USA) and LC-MS. The GS-MS was equipped with a flame ionization detector and capillary column (30 m length × 0.25 mm ID coated with 5% phenyl 95% dimethylpolysiloxane) with a film thickness of 0.25 μm. Helium gas used as mobile/carrier gas was operated at flow rate of 1 mL/min. During operation, the temperature of the injection port was kept at 280 °C, and analysis was carried out with 1 μL injection volume. The temperature of stationary phase was at 60–300 °C. The mass spectrum was prepared with full scan mode considering the range of 40–450 Daltons [49]. LC-MS profile was studied with Agilent 6400 (Santa Clara, CA, USA) Series Triple Quadrupole System equipped with an electrospray ionization (ESI) source. The separation was done with Phenomenex Luna PFP analytical column at 40 °C at with injection volume of 10µL. For LC analysis the column temperature was 40 °C and a combination of 0.1% aqueous solution of formic acid and methanol (eluent A and B ratio was 80:20) was used as mobile phase at a flow rate of 0.40 mL/min under isocratic elution conditions. The obtained peak was identified with the standard libraries from NIST (The National Institute of Standards and Technology) library and Wiley. 

### 4.10. Molecular Docking Studies

The structures of phytochemicals and breast cancer receptor proteins 3CQW (Akt1), 3PP0 (HER2/ERBB2), and 5F1A (COX2) were downloaded from PubChem and PDB, respectively. Docking analysis was conducted using AutoDock Tools (ADT) [58,59]. The control drugs used for docking studies were AFT and LTB (Akt1), PTB and NTB (HER2), and ASP and PCM (COX2). UCSF Chimera and PyMoL were used for viewing molecules. Discovery Studio viewer was used for viewing amino acid interactions with docked compounds.

## 5. Conclusions

Breast cancer is one of the major causes of mortality and morbidity in women around the globe and may be triggered by multiple causes, including inflammation and oxidative stress, and microbial infection is one of the factors lead to inflammatory responses. Both oxidative stress and inflammation cause diverse ailments. Tackling multiple symptoms is a challenge for effective treatment, and there is an urgent need for safer and multifunctional drugs. Biomolecules have been considered safer and effective than synthetic drugs. Plants are the biggest source of bioactive molecules that might serve as potential lead compounds. *P. dicoccos* has been exploited in ancient literature and in the current work; its leaves have been considered for evaluation owing to antimicrobial, antioxidant, anti-inflammatory, and anti-cancer potential of this plant against breast cancer cells. In comparison to standard antibiotics, plant extract was not too effective but the anti-inflammatory and antioxidant profile is compatible. In addition, leaf extract was effective against MCF-7 breast cancer cells and have IC_50_ value of 34 μg/mL. In silico analysis is preferred to study the efficiency of compounds responsible for cancer inhibition. In silico assessment against selective receptors involved in cancer progression have suggested the efficiency and interaction of phytochemicals against specific biological targets. The efficiency and potency of phytochemicals can be improved when purified and used in combination with other compounds for preparing drug formulations. The present study underlined the importance of plant-based compounds in treating ailments such as breast cancer without affecting normal cells. Further studies with purified components are necessary to establish the mechanisms of action of phytochemicals studied in this work. 

## Figures and Tables

**Table 1:** Docking results and interaction analysis of selected phytochemicals against Akt1, COX2, and HER2.

Targets	Types of Analysis	Ligands	ΔG (kcal/mol)	Ligand Efficiency	K_i_ (μM)
3CQW chain A (Akt1)	GC-MS	MST	−6.62	−0.47	14.03
		OBZ	−6.29	−0.37	24.53
		DHB	−5.65	−0.63	72.8
	LC-MS	KMF	−7.2	−0.34	5.29
	Control drugs	AFT	−9.36	−0.28	0.138
		LTB	−10.02	−0.25	0.045
5F1A Chain A (COX2)	GC-MS	MST	−7.86	−0.56	1.75
		OBZ	−6.32	−0.37	23.46
		DHB	−5.74	−0.64	62.3
	LC-MS	KMF	−6.75	−0.32	11.23
	Control drugs	ASP	−5.39	−0.41	112.58
		PCM	−6.41	−0.55	37.59
3PP0 Chain A (HER2)	GC-MS	OBZ	−7.49	−0.44	3.24
		MST	−7.39	−0.53	3.8
		DHB	−5.05	−0.56	198.31
	LC-MS	KMF	−6.9	−0.33	8.78
	Control drugs	PTB	−9.5	−0.23	0.109
		NTB	−9.51	−0.24	0.107

AFT—Afatinib; ASP—Aspirin; DHB—2,3-Dihydro-benzofuran; KMF—Kaempferol; LTB—Lapatinib; MST—Megastigmatrienone; NTB—Nilotinib; OBZ—2-Octylbenzoate; PCM—Paracetamol; PTB—Pyrotinib. ΔG—Binding energy; K_i_—Inhibition constant.

**Table 2:** Types of interactions and involved residues of Akt1 receptor with phytochemicals.

Types of Interaction	3CQW (Akt-1)					
	OBZ	MST	DHB	KMF	LTB	AFT
Conventional hydrogen bond/Carbon–hydrogen bond	-	Ala230	Leu213	Glu228, Ala230, Glu198, Leu156	Phe161, Glu234, Asp439, Asp292	Gly159, Asp292, Lys156, Glu284, Gly157, Asp439
Van der waals	Glu228, His207, Leu210, Arg206, Leu213, Leu202, Thr211, Ser205	Tyr229, Glu228, Thr211, Thr291, Asp292, Glu278, Glu234, Asn279, Phe438	His207, Tyr474, Leu210, Thr211, Leu202, Gln203	Leu202, Gly157, Thr211, Lys179, Tyr229, Tyr229	Glu191, His194, Gly294, Asn279, Asn274, Gly162, Lys163, Val164, Lys158, Gly157, Gly159, Leu156, Thr160, Glu278, Lys276, Phe237	Lys163, Thr291, Leu156, Phe237, Tyr437, Gly233, Phe442, Phe236, Glu278
Salt bridge/Attractive charge/Pi-lone pair/Pi-anion/Pi-sulfur Halogen bond	Lys419, Lys289	-	Ser205	Asp292, Met227	Phe438, Phe442	Met281, Gly162
Pi-stacked/Pi-Tshaped/Alkyl/Pi-alkyl	Ala212, Pro208	Met227, Ala177, Val164, Leu156, Met281	Ala212, Arg206		Leu295	Val164, Phe438, Lys179
Unfavorable Donor-Donor	-	-	-	Phe293	-	-

**Table 3:** Types of interactions and involved residues of COX-2 receptor with phytochemicals.

Types of Interaction	5F1A (COX-2)					
	OBZ	MST	DHB	KMF	ASP	PCM
Conventional hydrogen bond/Carbon–hydrogen bond/Pi-donor hydrogen bond	-	-	Trp387, His388	Lys511, Glu510, Glu520	His207, Tyr385, Trp387	Ala199, Tyr385, His207
Van der waals	Tyr475, Glu510, Gly519, His90, Thr94, His95, Pro512, Asn87	Phe207, Tyr385, Gln203, His388, Phe200	Ala199, Gln203, Tyr385, His386	-	Gln203, His388, Leu390, Ala202, Thr206, Thr386	Phe200, Leu391, His388, Glu203
Salt bridge/Attractive charge/Pi-lone pair/Pi-anion/Halogen bond	Lys511, Glu520	-	-	-	-	-
Pi-stacked/Pi-Tshaped/Alkyl/Pi-alkyl/Unfavorable acceptor-Acceptor	Arg518, Pro514, Tyr91	His207, His286, Ala202, Leu390, Ala199, Trp387, Leu391, Thr391	Leu391, Leu390, Ala202	Arg513	Leu391	His386, Leu390, Ala202

**Table 4:** Types of interactions involved and residues of HER2 receptor with phytochemicals.

Types of Interaction	3PP0 (HER-2)					
	OBZ	MST	DHB	KMF	NTB	PTB
Conventional hydrogen bond/Carbon–hydrogen bond/Pi-donor hydrogen bond	Tyr877	Lys753	-	Thr798, Thr862, Glu770	Arg756, Asn850, Arg849	Arg849, Asp843, Asp863
Van der waals	Val884, Lys883, Glu766, Gly865, Ala763, Glu770	Thr793, Val797, Ile752, Ala751, Thr862, Asp863, Phe864, Glu770	Arg784, Ala771, Glu770, Thr798, Ser783, Thr862, Asp863	Ap863, Leu852, Ser783, Arg784	Thr798, Asp863, Gly729, Val884, Gly727	Ala730, Phe731, Lys383, Val884, Leu866, Leu807, Gly727, Gly804, Leu852, Thr862, Thr798, Leu796, Ser728, Asn850
Salt bridge/Attractive charge/Pi-lone pair/Pi-anion/Halogen bond	Lys762, Arg868, Arg844, Gly882	-	-	-	Ser728, Pro885, Lys883, Asp845	-
Pi-Sigma/Pi-stacked/Pi-Tshaped/Alkyl/Pi-alkyl/Unfavorable acceptor-Acceptor	Phe731, Ile767, Leu866, Ala879	Leu796, Met774, Ala771, Leu785	Phe864, Met774, Leu785	Met774, Phe864, Leu785, Leu796, Ala751, Val734, Ly753	Thr862, Ala730, Phe731, Lys753, Leu852, Val734, Lys805	Pro885, Leu726, Val734, Ala751, Lys758, Gly729

## Data Availability

Not applicable.

## Supplementary Materials

The following supporting information can be downloaded at: https://www.mdpi.com/article/10.3390/molecules28207101/s1, Table S1: Qualitative phytochemical screening of *P. dicoccos* leaf extracts; Table S2: Quantitative analysis of methanolic *P. dicoccos* leaf extract; Table S3: Anti-bacterial activity profiling of methanolic *P. dicoccos* leaf extract; Table S4: GC-MS study of methanolic *P. dicoccos* leaf extract; Table S5: LC-MS study of methanolic *P. dicoccos* leaf extract; Figure S1: *P. dicoccos* botanical sample (Specimen #VK001).
