# Supplementary material for: Chemical Profiling and Biological Activity of Psydrax dicoccos Gaertn
Source: Molecules. 2023 Oct 15;28(20):7101. doi: 10.3390/molecules28207101 (PMC10609380; doi:10.3390/molecules28207101)
Supplement: Supplementary file 1 [file molecules-28-07101-s001.zip › molecules-2638640-supplementary.pdf]

Supplementary Materials

Chemical Profiling and Biological Activity of *Psydrax dicoccos* Gaertn

Kamaraj Veeramuthu [1], Vishal Ahuja [2,3], Pushparaj Annadurai [4], Daniel A. Gideon [5], Balamurugan Sundarrajan [1], Marius Emil Rusu [6,*], Vinothkanna Annadurai [7] and Kandavel Dhandayuthapani [8,*]

1 Thanthai Periyar Government Arts and Science College (Autonomous), Affiliated to Bharathidasan University, Tiruchirappalli 620023, Tamil Nadu, India
2 University Institute of Biotechnology, Chandigarh University, Mohali 140413, Punjab, India
3 University Centre for Research & Development, Chandigarh University, Mohali 140413, Punjab, India
4 C.P.R. Environmental Education Center, 1 Eldams Road, Alwarpet, Chennai 600018, Tamil Nadu, India
5 Department of Biochemistry, St. Joseph College, Bangalore 560025, Karnataka, India
6 Department of Pharmaceutical Technology and Biopharmaceutics, Faculty of Pharmacy, Iuliu Hatieganu University of Medicine and Pharmacy, 400012 Cluj-Napoca, Romania
7 School of Food and Biological Engineering, Jiangsu University, Zhenjiang 212013, China
8 Department of Botany, Government Arts College for Men, Nandanam, Affiliated to University of Madras, Chennai 600035, Tamil Nadu, India
* Correspondence: rusu.marius@umfcluj.ro (M.E.R.); kandavel1976@gmail.com (K.D.)

Table S1. Qualitative phytochemical screening of *P. dicoccos* leaf powder extracts.

Test	Aqueous	Methanol	Ethanol	Acetone	Chloroform	Hexane
Triterpenoid	+	+	+	+	-	+
Sugar	+	+	-	+	-	-
Flavonoid	-	+	+	-	+	-
Catachin	-	-	-	-	-	-
Saponin	+	-	+	-	-	-
Tannin	+	+	+	+	-	+
Anthraquinone	-	-	-	-	-	-
Amino acid	+	+	+	+	+	+
Sterol	+	+	-	-	+	+
Carbohydrate	+	+	+	+	+	-

Table S2. Quantitative analysis of methanolic *P. dicoccos* leaf extract.

Test	Amount of compounds present (mg/g)
Chlorophyll a	8.07 ± 0.2
Chlorophyll b	10.45 ± 0.1
Total chlorophyll	14.48 ± 0.4
Total carotenoids	2.18 ± 2.4
Total sugar	257 ± 0.2
Total protein	1.2 ± 1.1
Total lipids	182 ± 2.0
Total free amino acids	0.9 ± 0.1
Total phenolics	81.11 ± 1.1
Total tannin	51.09 ± 2.2
Total flavonoids	159 ± 2.8

Table S3. Anti-bacterial activity profiling of methanolic *P. dicoccos* leaf extract.

Antibiotic (µg/disc)	Zones represented as radius (mm)						
	Leaf extract conc. (mg/mL)	*E. coli*	*P. aeruginosa*	*S. aureus*	*S. flexneri*	*K. pneumoniae*	*S. typhimurium*
-	6	1.2±0.609	0.3±0.795	-	0.4±0.410	0.4±0.193	-
-	7	0.7±0.552	0.3±0.728	-	0.3±0.447	0.6±0.217	-
-	8	1.2±0.453	0.7±0.704	0.0±0.332	1.2±0.456	0.3±0.234	-
-	9	1.1±0.537	0.4±0.757	0.9±0.270	0.7±0.297	0.3±0.259	-
-	10	1.9±0.611	2.3±0.757	0.4±0.038	0.6±0.225	0.6±0.287	-
Amikacin (10 µg)	-	12±2.1	13±1.3	10±1.2	11±0.8	12±1.8	13±1.5
Chloramphenicol (10 µg)	-	11±2.1	6±1.3	3±1.0	4±1.8	5±1.8	8±0.8
Bacitracin (10 µg)	-	NE	NE	NE	NE	NE	NE
Penicillin G (30 µg)	-	NE	NE	NE	NE	NE	NE
Methicillin (5 µg)	-	NE	NE	NE	NE	NE	NE

NE – not effective. *Escherichia coli* (*E. coli*); *Pseudomonas aeruginosa* (*P. aeruginosa*); *Staphylococcus aureus* (*S. aureus*); *Shigella flexneri* (*S. flexneri*); *Klebsiella pneumoniae* (*K. pneumoniae*); *Salmonella typhimurium* (*S. typhimurium*).

Table S4. GC-MS study of methanolic *P. dicoccos* leaf extract.

Peak	Retention time	Name	Area	Retention Index	Molecular formula	Molecular weight	Docking results
1	11.603	Benzenecarboxylic acid	38374943	0	$C_7H_6O_2$	122	-
2	12.049	2,3-Dihydro-Benzofuran	712375	1036	C_8H_8O	120	+
3	14.13	2-Methoxy-4-Vinylphenol	804363	0	$C_9H_{10}O_2$	150	-
4	19.952	4-Methyloxy-6-methylcoumarin	939973	0	$C_{11}H_{10}O_3$	190	-
5	20.078	Cyclopenta[C]Pyran-4-Carboxylic Acid, 7-Methyl-, Methyl Ester	13844412	1402	$C_{11}H_{10}O_3$	190	-
6	20.71	Megastigmatrienone	1655818	1454	$C_{13}H_{18}O$	190	+
7	21.09-21.464	1,3,4,5-Tetrahydroxycyclohexanecarboxylic Acid	8979051	0	$C_7H_{12}O_6$	192	-
10	21.954	3,4-Dihydrocoumarin, 7,8-dimethyl-	4580894	1618	$C_{11}H_{12}O_2$	176	-
11	22.075	(E)-2,6-Dimethoxy-4-(prop-1-en-1-yl)phenol	2247222	0	$C_{11}H_{14}O_3$	194	-
12	22.41	1H,3H-Pyrano[3,4-c]pyran-1-one, 5-ethenyl-6-(.beta.-D-glucopyranosyloxy)-5,6-dihydro-, (5R-trans)-	9452624	3042	$C_{16}H_{20}O_9$	356	-
13	22.603	Mome Inositol	9435268	1647	$C_7H_{14}O_6$	194	-
14	22.81	(E)-4-(3-Hydroxyprop-1-en-1-yl)-2-methoxyphenol	4232584	1653	$C_{10}H_{12}O_3$	180	-
15	23.202	Tetradecanoic Acid	772831	1769	$C_{14}H_{28}O_2$	228	-
16	24.533	Neophytadiene	1078998	0	$C_{20}H_{38}$	278	-
17	24.95-25.265	3,7,11,15-Tetramethyl-2-hexadecen-1-ol	290870	2045	$C_{20}H_{40}O$	296	-
19	26.029	Methyl palmitate	1002064	1878	$C_{17}H_{34}O_2$	270	-
20	26.298	17-Pentatriacontene	467597	3508	$C_{35}H_{70}$	490	-
21	26.67	n-Hexadecanoic acid	7886533	1968	$C_{16}H_{32}O_2$	256	-
22	28.698	Linoleic acid	489690	2093	$C_{19}H_{34}O_2$	294	-
23	28.79	9,12,15-Octadecatrienoic acid, methyl ester, (Z,Z,Z)-	1458002	2101	$C_{19}H_{32}O_2$	292	-
24	28.964	Phytol	753451	2045	$C_{20}H_{40}O$	269	-
25	29.321	9,12-Octadecadienoic acid (Z,Z)-	2128485	2183	$C_{18}H_{32}O_2$	280	-
26	29.431	Dichloroacetic acid	9177045	2042	$C_{15}H_{24}Cl_2O_2$	306	-
27	29.773	Octadecanoic acid	2242046	2167	$C_{18}H_{36}O_2$	284	-
28	31.012	2-Oxa-3-azabicyclo[2.2.2]oct-5-ene, 3-benzoyl-	1262046	1755	$C_{13}H_{13}NO_2$	215	-
29	31.861	2-Octyl benzoate	955456	1692	$C_{15}H_{22}O_2$	234	+
30	34.602	Hexadecanoic acid, 2-hydroxy-1-(hydroxymethyl)ethyl ester	1836502	2498	$C_{19}H_{38}O_4$	330	-

31	34.881	Oxirane, hexadecyl-	399182	1901	$C_{18}H_{36}O$	268	-
32	36.923	Linolenic acid	1773973	2721	$C_{21}H_{36}O_4$	352	-
33	37.203	Stearic acid	888524	2681	$C_{21}H_{42}O_4$	358	-
34	38.32	Squalene	4467855	2914	$C_{30}H_{50}$	410	-
35	39.984	delta-Tocopherol	992557	2923	$C_{27}H_{46}O_2$	402	-

Table S5. LC-MS study of methanolic *P. dicoccos* leaf extract.

M/Z ratio	Compounds	Retention Time	Retention Index	Docking studies
160.9	Caffeoyl-O-hexoside	16.93	58	–
162.9	5-O-p-coumaroylshikimic acid (5-p-CoSA)	21.13	45	–
169	Quercetin	35.84	62	–
191.1	E)Catechin–(E)Gallocatechin a	27.56	41	–
195.8	Rhamnosylhexosyl luteolin	24.21	54	–
197.8	Kaempferol	26.65	32	+
199	4-O-p-coumaroylshikimic acid (3-p-CoSA)	22.92	86	–
274.3	5-O-p-coumaroylshikimic acid (5-p-CoSA)	21.13	42	–
377.2	Quercetin rutinoside	28.9	59	+
593.3	Rhamnosylhexosyl methyl quercetin	24.67	76	–
696.4	(E)Catechin–(E)Catechin–(E)Catechin–(E)Catechina	30.54	59	–
717.3	4-O-p-coumaroylshikimic acid (3-p-CoSA)	22.92	70	–
740.4	(E)Catechin–(E)Catechin–(E)Catechin–(E)Catechina	30.54	83	–

761.4	(E)-3-(3,4-diacetoxy-5-methoxyphenyl)acroyl-4-O-p-coumaroyl-5-O-caffeoylquinic acid	37.5	74	–
849.4	(E)Catechin–(E)Catechin–(E)Catechin a	21.42	48	-

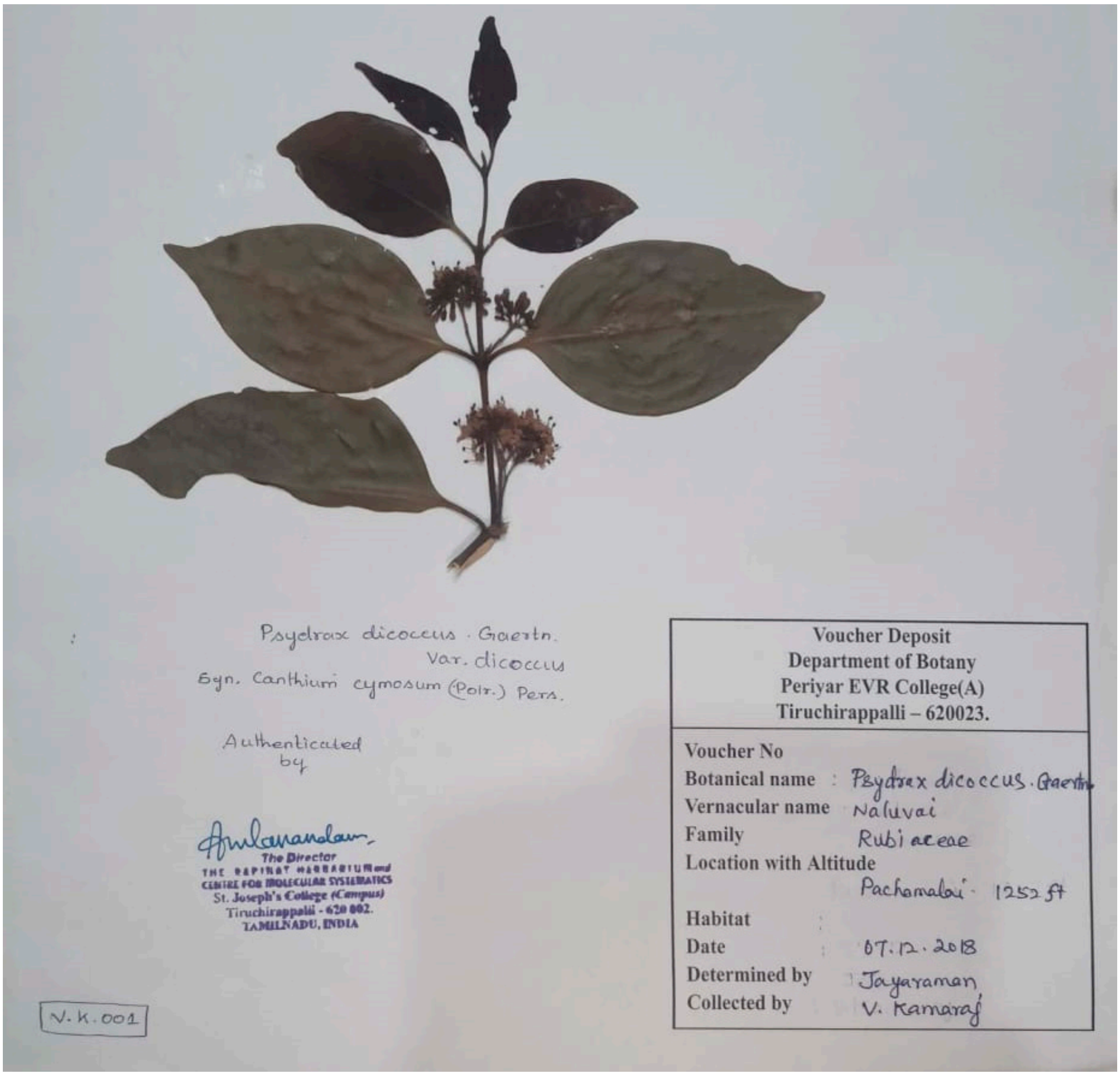

Figure S1. *P. dicoccos* botanical sample (Specimen #VK001).
